# Ranging Consistency Based on Ranging-Compensated Temperature-Sensing Sensor for Inter-Satellite Link of Navigation Constellation

**DOI:** 10.3390/s17061369

**Published:** 2017-06-13

**Authors:** Zhijun Meng, Jun Yang, Xiye Guo, Yongbin Zhou

**Affiliations:** College of Mechatronics Engineering and Automation, National University of Defense Technology, Changsha 410073, China; mzj727@126.com (Z.M.); john323@163.com (J.Y.); michaelzhou@nudt.edu.cn (Y.Z.)

**Keywords:** ranging consistency, temperature-sensing sensor, ranging compensation, inter-satellite link, navigation constellation

## Abstract

Global Navigation Satellite System performance can be significantly enhanced by introducing inter-satellite links (ISLs) in navigation constellation. The improvement in position, velocity, and time accuracy as well as the realization of autonomous functions requires ISL distance measurement data as the original input. To build a high-performance ISL, the ranging consistency among navigation satellites is an urgent problem to be solved. In this study, we focus on the variation in the ranging delay caused by the sensitivity of the ISL payload equipment to the ambient temperature in space and propose a simple and low-power temperature-sensing ranging compensation sensor suitable for onboard equipment. The experimental results show that, after the temperature-sensing ranging compensation of the ISL payload equipment, the ranging consistency becomes less than 0.2 ns when the temperature change is 90 °C.

## 1. Introduction

Position, velocity, and time accuracy, along with integrity, continuity, and availability, is the four major performance indicators of satellite navigation systems [1]. Navigation system innovations and enhancements require technical advancements. Among the many techniques for improving the Global Navigation Satellite System (GNSS) performance, inter-satellite link (ISL) technology has become a research focus in recent years. National Aeronautics and Space Administration Senior Engineer K. P. Maine demonstrated the GPS III ISL. He stated that the introduction of ISLs can significantly improve the ephemeris prediction accuracy, realize autonomous integrity monitoring of satellites, increase autonomous orbit determination, and improve navigation system flexibility and expansibility [2]. Russia’s new generation of navigation satellite GLONASS (GLObalnaya NAvigatsionnaya Sputnikovaya Sistema) -M also planned to improve autonomous navigation using ISLs [3,4]. The European Space Agency has initiated several exploratory projects on the ISLs adopted in the Galileo system since 2007 [5]. Recently, with the successful launch of the new generation BeiDou (Xichang, China) navigation networking satellites, ISLs have been used in the BeiDou navigation system to achieve autonomous orbit determination [6,7]. The introduction of ISLs has marked the emergence of a new generation of satellite navigation systems. Construction of ISLs has become an important consensus in several current GNSSs [8,9].

The accuracy of inter-satellite ranging is crucial for determining the quality of the ISL construction, and ranging consistency is a prerequisite for ranging accuracy. M. P. Ananda set the precision of inter-satellite ranging to 0.5 m to simulate autonomous orbit determination [10]. Song used ISL and seven ground monitoring stations in China to achieve joint orbit determination. When the ISL pseudo-range observation error was 0.8 m, the orbit accuracy was better than 0.7 m [11]. Moreover, Tang used ISL measurements in a centralized mode of the new-generation BeiDou navigation satellites to conduct autonomous orbit-determination experiments. When the standard deviation of the ISL measurement residuals was within 10.0 cm, the radial overlap differences in the autonomous orbits were less than 6.0 cm. The 24-h predicted orbital radial overlap differences were less than 10.0 cm, and the user equivalent ranging error of the 24-h predicted autonomous orbits was approximately 0.43 m [12]. According to the present notion of autonomous orbit determination and satellite-ground joint orbit determinations, the accuracy of inter-satellite ranging should be on the decimeter level.

To date, most studies have focused on the ISL signal system design [13], signal dynamic characteristic analysis [14,15], constellation design [16], and extension application [17,18]. Few works focus on ISL ranging consistency in navigation constellation. Ranging consistency is affected by the onboard clock equipment, ISL payload device, space environment, source transmitter, etc. Ref. [19] focused on the influence of the frequency and phase characteristics of the onboard clock equipment on the ranging consistency of the ISL and proposed a phase compensation sensor design method for the phase offset of the onboard clock equipment. In addition, the ISL payload device delay is mainly affected by the device aging, light deformation, day and night temperature difference, and other space environmental factors. Device aging and light deformation have a long-term effect on the process, and the effect of intensity is very small. However, a nanosecond level of delay variations mainly caused by temperature variations is possible [20] and the influence of temperature on the equipment time-delay could not be ignored for high-precision satellite-ranging [21]. The temperature difference between sun-lighted and shaded areas can reach up to 200 °C. Meanwhile, the general navigation satellite payload thermal-control design range is approximately 40 °C, and the actual temperature difference in the payloads may exceed this range. Therefore, in the present study, we focus on the variation in the ranging delay caused by the sensitivity of the ISL payload equipment to the ambient temperature in space and propose a simple and low-power temperature-sensing ranging compensation sensor suitable for onboard equipment.

The next section describes the ranging principle of ISL payload, the composition of the ISL payload, the operating environment, and the relevant temperature-dependent components. Section 3 presents the design approach for ranging-compensated temperature-sensing sensor. The field data collection and ranging experiments are described in Section 4. The final section presents our conclusions.

## 2. Analysis of Ranging Consistency for ISL Payload

### 2.1. Ranging Principle of ISL Payload

The dual-one-way measurement of an ISL consists of two satellites alternately performing a pseudo-code phase ranging process and the mutual transfer of the measurement results. It contains two one-way pseudo-range measurements.

**Figure 1:**
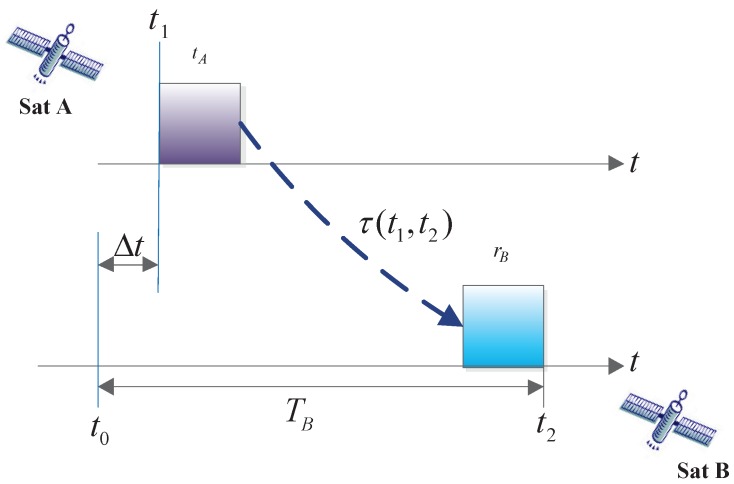
One-way pseudo-range measurement process.

As shown in Figure 1,

(1) $$T_{B}\;=\;\Delta t+t_{A}+\tau \left(t_{1},t_{2}\right)+r_{B}=t_{2}-t_{0},$$

where $T_{B}$ is the measurement pseudo-range, Δt is the system clock difference between satellite *A* and satellite *B*, $t_{A}$ is the ISL payload transmitting circuit delay of satellite *A*, $r_{B}$ is the ISL payload receiving circuit delay of satellite *B*, $\tau (t_{1},t_{2})$ is the spatial delay, $t_{1}$ is the transmitting point time of satellite *A*, and $t_{0}$ and $t_{2}$ are the reception point time and the measurement point time of satellite *B*, respectively. In the current study, we focus on the variation in the ranging delay, namely, $t_{A}$ and $r_{A}$ and $t_{B}$ and $r_{B}$, caused by the sensitivity of the ISL payload transmission and reception circuits to the ambient temperature in space. Next, we describe the composition of the ISL payload transmission and reception circuit.

### 2.2. Overview of ISL Payload Composition and Delay Distribution

**Figure 2:**
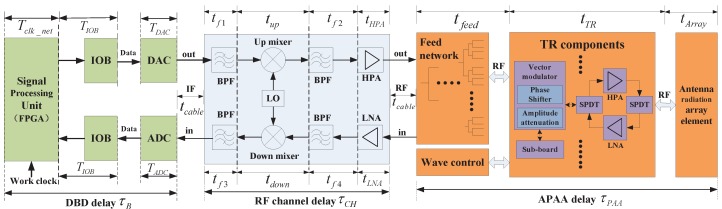
Inter-satellite link (ISL) payload principle, architecture, and related delay distribution.

The ISL of a navigation constellation adopts a space- and time-division multiplexing access system. The navigation satellite based on the planning table of the entire network link is associated with different visible satellites in different slot divisions. The antenna beam should perform constant fast switching; thus, the beam agile active phased-array antenna (APAA) has become the first choice for a global ISL network. The ISL transceiver is specific payload equipment for inter-satellite ranging and communication. It consists of a digital baseband (DBD) signal-processing module and an radio frequency (RF) transceiver channel. The baseband module performs spread-spectrum signal processing and algorithm protocol implementation. The RF transceiver channel realizes the signal up-down conversion and power amplification. The ISL payload principle, architecture, and related delay distribution are shown in Figure 2.

In Figure 2, IF represents the intermediate-frequency interconnection, and RF represents the radio-frequency interconnection. In DBD, IOB represents the input and output block, DAC represents the digital-to-analog converter, and ADC represents the analog-to-digital converter. In the RF channel, BPF denotes the band-pass filter, LO denotes the local oscillator, HPA represents the high-power amplifier, and LNA represents the low-noise amplifier. In APAA, SPDT represents the single-pole double-throw switch, TR denotes the transmitter/receiver, and HPA and LNA have the same meaning as those in the RF channel.

### 2.3. Temperature Characteristic of the ISL Payload

Because of the existence of nonlinear phase units in the RF channel and APAA, such as the frequency converter, amplifier, LNA, and filter, the temperature changes generate a group delay and phase distortion when the spread-spectrum signals pass through these non-ideal transmission channels. In addition, the field-programmable gate array (FPGA), ADC, DAC, buffer, and other digital components in the DBD generate time-delay changes with the change in temperature. These changes with the temperature fluctuations are included in the pseudo-code ranging measurements of the distance. The delay characteristics of these equipment components affected by the temperature are inconsistent. Some increase with temperature in which the delay becomes smaller, such as the antenna feed network [22], whereas others increase with temperature in which the delay becomes larger, such as the frequency up-converter [23]. Because almost no information is available from manufacturers on the sensitivity of the group delay to environmental parameters of the component used in a two-way satellite time and frequency transfer (TWSTFT) device, Ref. [24] measured the values (or range of values) and uncertainties of the temperature coefficients of the group delay observed from a variety of modules used in typical TWSTFT systems, including the LNA, HPA, filters, mixers, isolators, up converter, and down-converter, which are listed in Table 1.

Meanwhile, Ref. [24] measured the temperature coefficients of the delays of the whole transmission and reception channels of the TWSTFT. The temperature coefficient reaches up to 230 ps/°C. Ref. [25] showed similar statistics for the delay equipment. The delay in the ISL payload component also demonstrated such temperature characteristics. Moreover, at different temperature points, the degree of delay variation is also inconsistent. The ISL antenna is mounted in an extravehicular manner on a satellite payload, and the RF channel and DBD are installed in the cabin. Obviously, the temperature of the whole ISL payload is unevenly distributed. The consistency delay error of the equipment introduced by temperature variation is a large error source in a two-way radio ranging system. If it is not controlled, the measurement error can reach up to tens of nanoseconds, which directly affects the key performance index of the ISL-ranging accuracy.

## 3. Proposed Ranging-Compensated Temperature-Sensing Sensor Design Method

### 3.1. Architectural Overview

**Figure 3:**
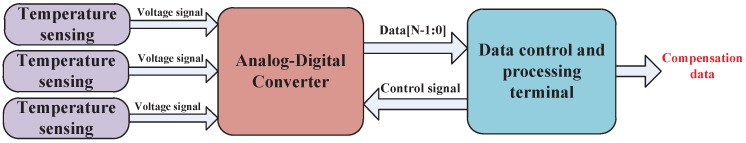
Architecture of the ranging compensation temperature-sensing sensor.

The architecture of the proposed ranging compensation temperature-sensing sensor is shown in Figure 3, which includes the temperature-sensing circuit, ADC, data control and processing terminal. The temperature-monitoring circuit converts the ambient temperature into an analog voltage signal. The ADC converts the collected voltage signal into a digital signal. The data control and processing terminal is responsible for controlling the multichannel ADC and converting the digital signal into transmitted and received delay compensation data of the ISL payload.

### 3.2. Data Preprocessing Flow and Method

The data preprocessing flow includes the data collection of temperature versus delay measurement, deviation delay data processing, compensation data storage, and compensation data calling. Each step involves a corresponding mathematical processing method, as described in details below.

We assume that the number of sampling bits of the ADC is *N* and the ambient temperature range in space is ΔT. Then, the temperature resolution is $\frac{\Delta T}{2^{N}}$. We also assume that the sampling rate of the ADC is *S* Hz and the sampling value is $x_{n}$. Because the space-temperature change is a gradual process, to avoid acquisition of instantaneous noise by the ADC resulting in compensation data bias, we first integrate sampling data $x_{n}$. The integration time is *t*. The starting point for the integration is the transmission or reception moment of the ISL payload ranging signal, and *t* is much smaller than the time of ambient temperature change $\frac{\Delta T}{2^{N}}$. Then, we calculate the arithmetic mean of the integral result and obtain sample value *V*. Traversing the entire temperature range can provide sequence $V_{i}$, which is expressed as follows:

(2) $$V_{i}=\frac{\sum_{n=0}^{St}x_{n}}{St}\;\;\;\;,\;\;n\in \left[0,\;\;2^{N}-1\right].$$

Each $V_{i}$ corresponds to transmission-delay value $\tau_{i}^{T}$ and reception-delay value $\tau_{i}^{R}$. Because the ground temperature-control equipment cannot achieve precise temperature control with resolution of $\frac{\Delta T}{2^{N}}$, measured sequence $V_{i}$ cannot traverse the data space $\left[0,2^{N}-1\right]$. Therefore, we need to use the MATLAB (R2014a, MathWorks, Natick, Massachusetts, USA) tool function polyfit(x,y,n) to perform data fitting for $\tau_{i}^{T}$ and $\tau_{i}^{R}$, which yields the following:

(3) $$\begin{array}{c} \left\{\begin{array}{c} \left[p^{T},S^{T}\right]=polyfit\left(V_{i},\tau_{i}^{T},k\right),\\ \left[p^{R},S^{R}\right]=polyfit\left(V_{i},\tau_{i}^{R},k\right), \end{array}\right. \end{array}$$

where *k* is the order of the fitting polynomial, $p^{T}$ and $p^{R}$ are the coefficients of the fitting polynomial, and structures $S^{T}$ and $S^{R}$ are used to obtain error estimates with function polyval(p,x). Then, we use function polyval(p,x) to determine fitting sequences $\tilde{\tau_{i}^{T}}$ and $\tilde{\tau_{i}^{R}}$, which are expressed as

(4) $$\begin{array}{c} \left\{\begin{array}{c} \left[\tilde{\tau_{i}^{T}},delta\right]=polyval\left(p^{T},V_{N},S^{T}\right),\;V_{N}=\left(V_{0},V_{1},V_{2},\cdots ,V_{2^{N}-1}\right),\\ \left[\tilde{\tau_{i}^{R}},delta\right]=polyval\left(p^{R},V_{N},S^{R}\right),\;V_{N}=\left(V_{0},V_{1},V_{2},\cdots ,V_{2^{N}-1}\right), \end{array}\right. \end{array}$$

where delta is the estimate of the standard deviation of the fitting error and $V_{N}$ is the sample sequence of the *N*-bit ADC. To ensure accuracy of the collected data, we keep the ISL payload connection state unchanged, traverse again the temperature change interval, and fit the test data *j* times. Then, we obtain transmission-delay matrix $\tilde{\tau_{i,j}^{T}}$ and reception-delay matrix $\tilde{\tau_{i,j}^{R}}$ as follows:

(5) $$\left\{\begin{array}{c} \tilde{\tau_{i,j}^{T}}=\left(\begin{array}{ccc} \tilde{\tau_{1,1}^{T}} & \ldots & \tilde{\tau_{1,j}^{T}}\\ \vdots & \ddots & \vdots \\ \tilde{\tau_{2^{N}-1,1}^{T}} & \cdots & \tilde{\tau_{2^{N}-1,j}^{T}} \end{array}\right)\\ \tilde{\tau_{i,j}^{R}}=\left(\begin{array}{ccc} \tilde{\tau_{1,1}^{R}} & \ldots & \tilde{\tau_{1,j}^{R}}\\ \vdots & \ddots & \vdots \\ \tilde{\tau_{2^{N}-1,1}^{R}} & \cdots & \tilde{\tau_{2^{N}-1,j}^{R}} \end{array}\right). \end{array}\right.\;$$

We sum up *j* times fitting sequences $\tilde{\tau_{i}^{T}}$ and $\tilde{\tau_{i}^{R}}$ and calculate their respective arithmetic means to obtain transmission time-delay fitting sequence $\bar{\tilde{\tau_{i}^{T}}}$ and reception time-delay sequence $\bar{\tilde{\tau_{i}^{R}}}$ corresponding to the final temperature range, i.e.,

(6) $$\left\{\begin{array}{c} \bar{\tilde{\tau_{i}^{T}}}=\frac{\sum_{n=1}^{j}\tilde{\tau_{i,n}^{T}}}{j}\\ \bar{\tilde{\tau_{i}^{R}}}=\frac{\sum_{n=1}^{j}\tilde{\tau_{i,n}^{R}}}{j} \end{array}\right.\;\;\;\;\;\;\;\;;\;\;\;\;\;\;\;i=\left(0,1,2\cdots ,2^{N}-1\right).$$

We assume that the ADC sampling value corresponding to the stabilization temperature in the satellite cabin is $V_{c}$. Then, $\bar{\tilde{\tau_{i}^{T}}}$ and $\bar{\tilde{\tau_{i}^{R}}}$, which correspond to $V_{c}$, are $\bar{\tilde{\tau_{c}^{T}}}$ and $\bar{\tilde{\tau_{c}^{R}}}$. The delay deviation sequence of the entire temperature range relative to $V_{c}$ in the satellite cabin is expressed as follows:

(7) $$\left\{\begin{array}{c} \Delta \tau_{i}^{T}=\bar{\tilde{\tau_{c}^{T}}}-\bar{\tilde{\tau_{i}^{T}}}\\ \Delta \tau_{i}^{R}=\bar{\tilde{\tau_{c}^{R}}}-\bar{\tilde{\tau_{i}^{R}}} \end{array}\right.\;\;\;\;\;\;\;\;;\;\;\;\;\;\;\;i=\left(0,1,2\cdots ,2^{N}-1\right).$$

We convert sequences $\Delta \tau_{i}^{T}$ and $\Delta \tau_{i}^{R}$ into pseudo-code delay phase values $\Delta P_{i}^{T}$ and $\Delta P_{i}^{R}$ and store them in the read-only memory (ROM) of the FPGA, which is the DBD data-processing chip. The read address of the ROM is ADC sample value $V_{i}$, and the read data are the delay compensation data. By assuming that the pseudo-code rate of the ISL ranging is $C_{V}$ and the bit width of the pseudo-code numerically controlled oscillator is *M*, we have

(8) $$\left\{\begin{array}{c} \Delta P_{i}^{T}=2^{M}\cdot \Delta \tau_{i}^{T}\cdot C_{V}\\ \Delta P_{i}^{R}=2^{M}\cdot \Delta \tau_{i}^{R}\cdot C_{V} \end{array}\right.\;\;\;\;;\;\;\;\;\;\;\;\;i=\left(0,1,2\cdots ,2^{N}-1\right).$$

**Figure 4:**
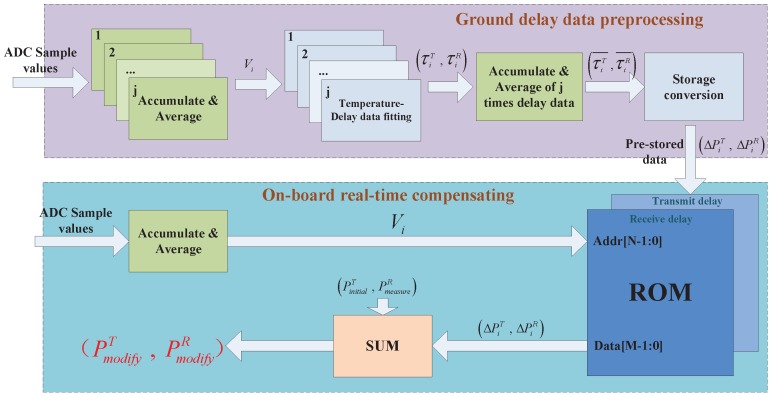
Entire data-processing diagram of the ranging compensation temperature-sensing sensor.

We need to open up a single block of $2^{N}\times M$ ROM capacity in the FPGA. All of these tasks are performed in advance on the ground. The onboard ISL payload only needs to read the compensation data at the time of transmission or reception as delay correction, where the read address is the integral result of the ADC sample value. When the ISL payload is received, modified ranging phase-delay value $P_{modify}^{R}$ is expressed as

(9) $$P_{modify}^{R}=P_{measure}^{R}+\Delta P_{i}^{R},$$

where $P_{measure}^{R}$ is the measured phase delay. When the ISL payload is transmitted, modified preset pseudo-code initial phase value $P_{modify}^{T}$ is expressed as

(10) $$P_{modify}^{T}=P_{initial}^{T}+\Delta P_{i}^{T},$$

where $P_{initial}^{T}$ is the initial preset pseudo-code transmission phase of the ISL payload. The diagram of the whole ranging compensation temperature-sensing sensor data processing is shown in Figure 4.

### 3.3. Circuit Design

**Figure 5:**
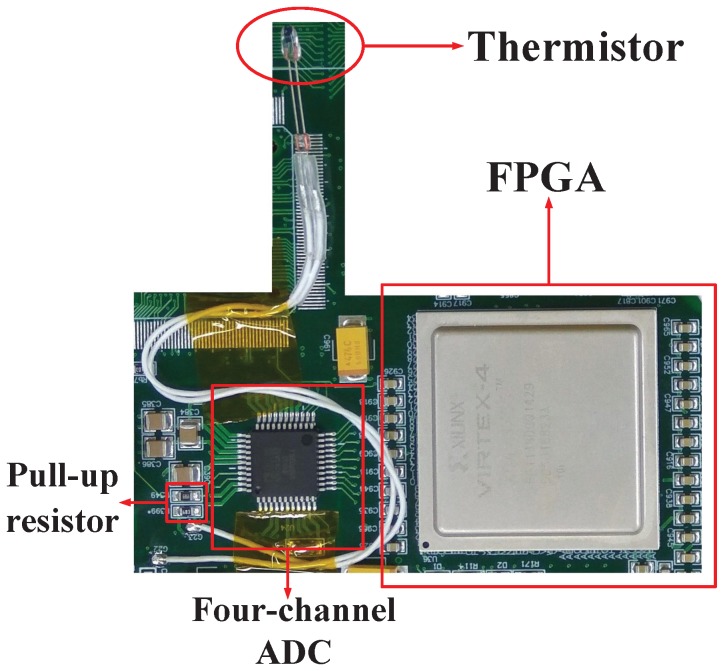
Design of the ranging compensation temperature-sensing sensor circuit.

On the basis of the design principle for simplicity, low power consumption, and adaptability to space environment, the design of the ranging compensation temperature-sensing sensor circuit is shown in Figure 5.

The temperature-sensing circuit converts the temperature into a voltage signal using a thermistor and a pull-up resistor. The thermistor can be distributed in the ISL payload of the APAA, RF channel, and DBD, and selection of the resistance value of the pull-up resistor is made according to the temperature-resistance range of the thermistor. To satisfy multi-module temperature sensing, the ADC uses the multi-channel simultaneous sampling chip AD7865, which is a 14-bit four-channel ADC. The data control and processing terminal uses the FPGA, which can be directly integrated into the ISL payload DBD processing chip. The complete temperature-sensing sensor can be integrated in the ISL DBD printed circuit board except the thermistor, and the occupied area is only 15 mm × 12 mm. The FPGA logic and storage resources only introduce six adders and six $2^{N}\times M$ ROM storage capacity, which is less than the Xilinx XQR4VSX55 (San Jose, California, USA) slice resource of 1%. In addition, the overall power-consumption increase is less than 90 mW.

## 4. Field Data Collection and Ranging Experiment

**Figure 6:**
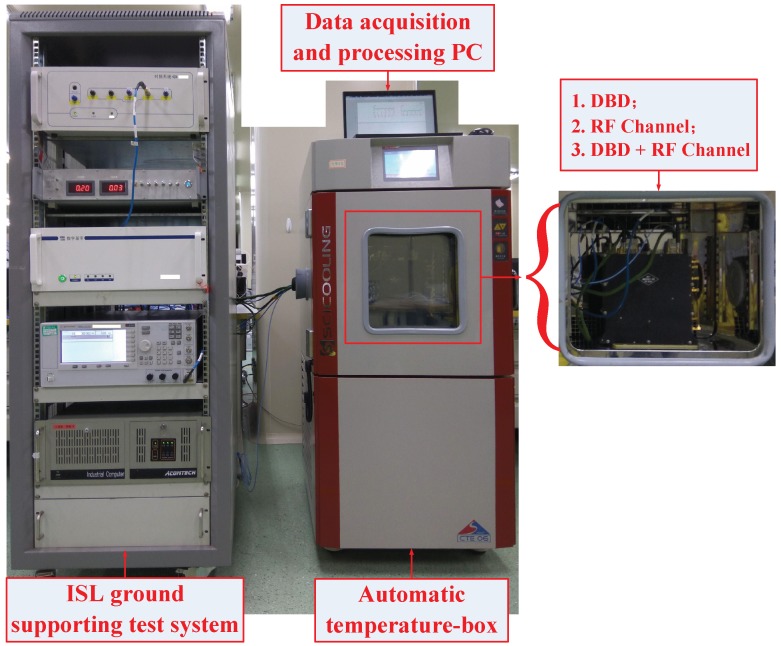
Test scenarios of the field data collection and ranging comparison.

To verify that the proposed circuit and method are valid, we used an engineering prototype for field data acquisition and ranging comparison. The selected thermistor in the −40–80 °C temperature range corresponded to a resistance range of 500–205 KΩ. Single-channel sampling rate *S* was set to 100 KSPS, and integration time *t* was 1 ms. The data control and processing terminal was integrated into the FPGA of the DBD. To facilitate the trial, we used an RF cable connection. The thermistors were installed in the ISL payload of the DBD and the RF channel and then put into the temperature box for data acquisition. The effective control of the temperature box is −30–60 °C, which is within the temperature tolerance of an industrial-grade chip. The rate of change in the temperature was 0.5 °C/min. The test scenario is shown in Figure 6.

**Figure 7:**
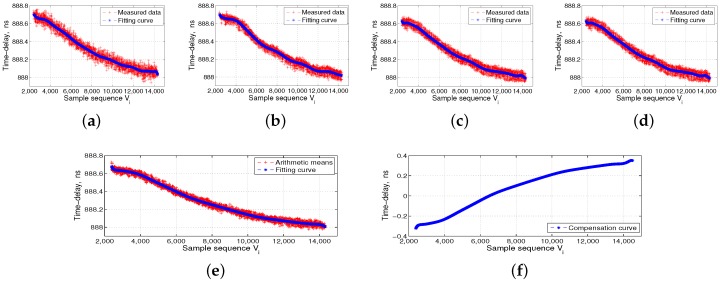
Digital baseband (DBD) transmission-delay measurement data preprocessing. (**a**–**d**) the transmission-delay data measured four times and the corresponding fitting curves; (**e**) the arithmetic means of the delay data measured four times and the corresponding fitting curves; and (**f**) the final transmission-delay compensation curves of the DBD.

**Figure 8:**
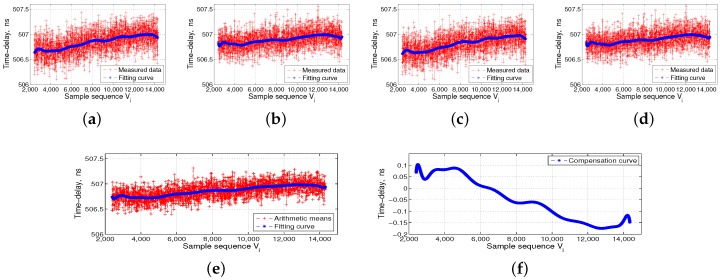
Digital baseband (DBD) reception-delay measurement data preprocessing. (**a**–**d**) the reception-delay data measured four times and the corresponding fitting curves; (**e**) the arithmetic means of the delay data measured four times and the corresponding fitting curves; and (**f**) the final reception-delay compensation curves of the DBD.

**Figure 9:**
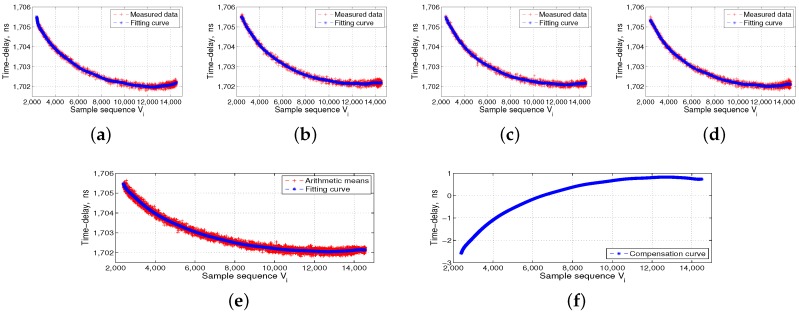
Radio frequency (RF) channel transmission-delay measurement data preprocessing. (**a**–**d**) the transmission-delay data measured four times and the corresponding fitting curves; (**e**) the arithmetic means of delay data measured four times and the corresponding fitting curves; and (**f**) the final transmission-delay compensation curves of the RF channel.

**Figure 10:**
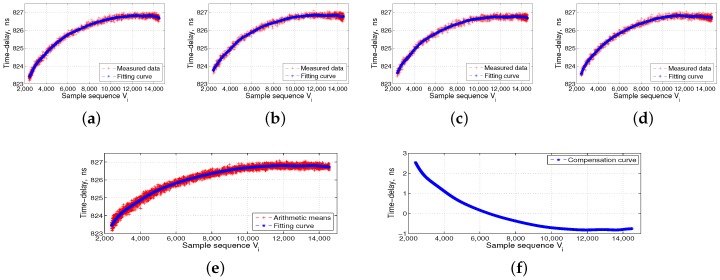
RF channel reception-delay measurement data preprocessing. (**a**–**d**) the reception-delay data measured four times and the corresponding fitting curves; (**e**) the arithmetic means of the delay data measured four times and the corresponding fitting curves; and (**f**) the final reception-delay compensation curves of the RF channel.

The left side of Figure 6 shows the ISL ground-support test system, which includes the time-frequency module, ground ISL signal transceiver of the baseband module, RF channel module, and industrial computer. The middle panel in Figure 6 shows the automatic temperature box, and the data acquisition and processing PC is located at the top of the temperature box. The right side of Figure 6 shows the ISL payload equipment, which is put into the temperature box, followed by the DBD, RF channel, and the baseband and channel as a unit. We separately collected four groups of transmitted and received data for the DBD and RF channel. The flow processes of 16 groups of measured data are shown in Figure 7, Figure 8, Figure 9 and Figure 10.

The temperature reference shown in Figure 7, Figure 8, Figure 9 and Figure 10 is 14 °C, i.e., $V_{c}$ is 6500. We set the order of fitting polynomial *k* equal to 20. Subpanels (a–d) in Figure 7, Figure 8, Figure 9 and Figure 10 show the delay data curves measured four times and the corresponding fitting curves. According to Equation (6), subpanel (e) shows $\bar{\tilde{\tau_{i}^{T}}}$ or $\bar{\tilde{\tau_{i}^{R}}}$, the arithmetic means of the delay data measured four times and the corresponding fitting curves. According to Equation (7), $\bar{\tilde{\tau_{c}^{T}}}$ and $\bar{\tilde{\tau_{c}^{R}}}$ corresponding to $V_{c}$ of 6500, and subpanel (f) shows $\Delta \tau_{i}^{T}$ or $\Delta \tau_{i}^{R}$, i.e., the final delay compensation curves. Figure 7 and Figure 8 show the DBD transmission- and reception-delay measurement data preprocessing, respectively. Figure 9 and Figure 10 show the RF channel transmission- and reception-delay measurement data preprocessing, respectively. Figure 7, Figure 8, Figure 9 and Figure 10 show that the DBD is less affected by temperature, and its transmission and reception delays change at the level of 1 ns. Meanwhile, the RF channel, because it contains more analog nonlinear components, is more affected by the temperature. Its transmission and reception delays change at the level of 5 ns.

**Figure 11:**
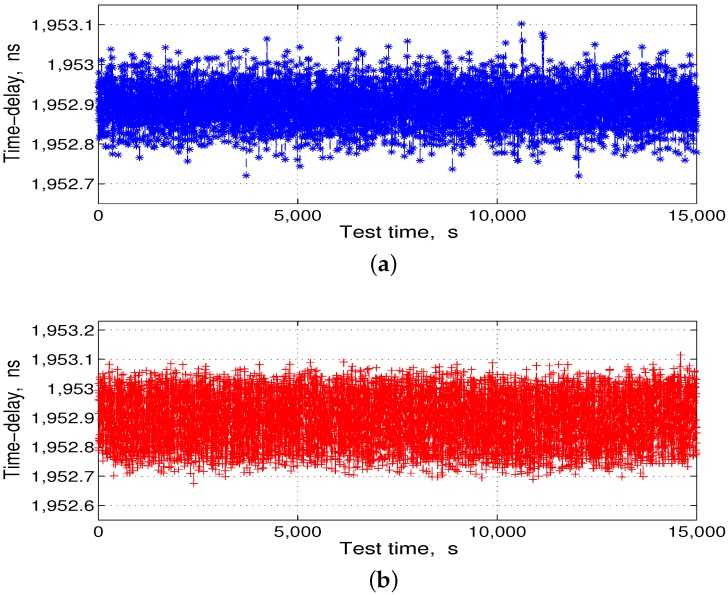
Transmission-delay compensation comparison test results. (**a**) test at 14 °C; and (**b**) test at −30–60 °C.

**Figure 12:**
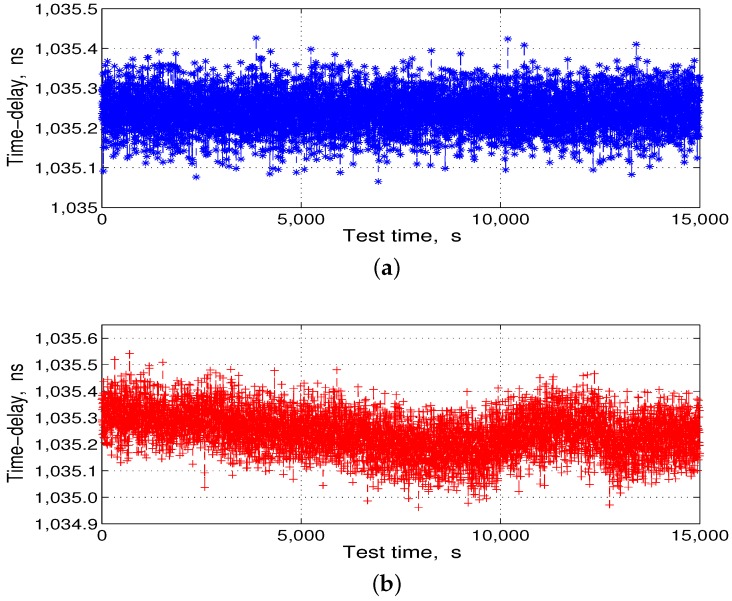
Reception-delay compensation comparison test results. (**a**) test at 14 °C; and (**b**) test at −30–60 °C.

After the data processing, from Equation (8), the corresponding converted temperature compensation data were stored in the ROM of the FPGA. Then, we put the DBD and the RF channel together in the temperature box and controlled the temperature box at 14 °C and −30–60 °C for the delay compensation comparison test. Each test time was longer than 4 h, and the test results are shown in Figure 11 and Figure 12.

**Figure 13:**
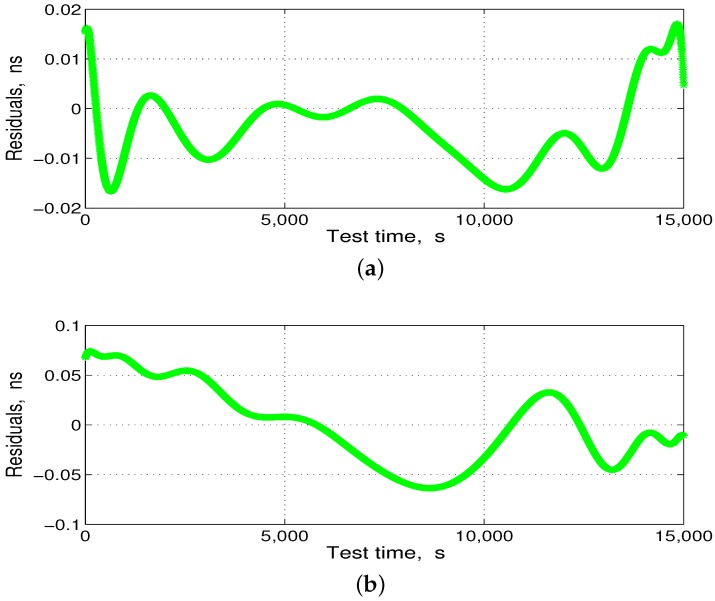
Delay residuals after compensation in the temperature-change test; (**a**) transmission-delay residuals; and (**b**) reception-delay residuals.

Subsequently, we subtracted the statistical mean shown in Figure 11a and Figure 12a from the delay-fitting value shown in Figure 11b and Figure 12b, respectively, to obtain the residuals, as shown in Figure 13.

Figure 13 shows that the delay residual error is less than that in the pseudo-code measurement. In other words, after the ranging compensation temperature-sensing sensor, the delay mean at 90 °C temperature changes reached a pseudo-code error of 0.2 ns, ensuring consistency in the ISL ranging.

## 5. Conclusions

In this study, we designed a ranging-compensated temperature-sensing sensor for ISL payload. Considering the hardware limitation of an onboard payload device, the proposed sensor only needs additional thermistors, a number of resistors, and a multi-channel ADC. The data-processing terminal can be integrated in the onboard processor FPGA, and the FPGA simply uses the ADC sampling value to read out the pre-processed compensation data. After the analysis of the experimental data, the proposed compensation circuit can control the temperature variation at a 5-ns level of delay error within 0.2 ns and ensure consistency in the ISL payload delay ranging.

## Figures and Tables

**Table 1:** Temperature coefficients (TC) of group delay for modules (measured at +5–+45 °C, 40% relative humidity).

Component	TC, Unit: ps/°C	Comments
HPA	−5 (±2)	Transmission path
LNA and filter	−5 to +2 (±2)	Reception path
Mixer and isolator	−18 (±2)	Transmission path or reception path
Up-Converters	−4 to +1 (±2)	Transmission path
Down-Converters	+2 to +10 (±2)	Reception path
